# Cross-lineage anomaly detection of microRNA expression: a framework for deriving exploratory candidate biomarkers in sarcoma

**DOI:** 10.3389/fgene.2026.1907450

**Published:** 2026-10-12

**Authors:** Mritunjoy Dey, Piotr Remiszewski, Ewelina Perdas, Paweł Golik, Jakub Piątkowski, Anna M. Czarnecka

**Affiliations:** 1 Department of Soft Tissue/Bone Sarcoma and Melanoma, Maria Sklodowska-Curie National Research Institute of Oncology, Warsaw, Poland; 2 Medical Faculty, Medical University of Warsaw, Warsaw, Poland; 3 Department of Biostatistics and Translational Medicine, Medical University of Lodz, Lodz, Poland; 4 Institute of Genetics and Biotechnology, Faculty of Biology, University of Warsaw, Warsaw, Poland; 5 Institute of Biochemistry and Biophysics Polish Academy of Science, Warsaw, Poland; 6 Department of Experimental Pharmacology, Mossakowski Medical Research Institute, Polish Academy of Sciences, Warsaw, Poland; 7 Outpatient Chemotherapy Department, Maria Sklodowska-Curie National Research Institute of Oncology, Warsaw, Poland

**Keywords:** biomarker prognosis, computational genomic analysis, mesenchymal identity, microRNA, sarcoma

## Abstract

**Introduction:**

Sarcomas are rare, heterogeneous malignancies of mesenchymal origin whose diagnosis and molecular classification remain challenging. We introduced a oneclass anomaly detection framework to microRNA expression profiles to test whether sarcoma-associated miRNA features are distinguishable from those of epithelial, haematological, and neurological cancers and if these features carry any biological relevance.

**Methods:**

Candidate miRNAs meeting cross-lineage significance criteria were further evaluated through differential expression analysis against subtype-matched normal mesenchymal reference tissue, pathway enrichment, and survival analysis. A five-miRNA index (“Index 5”), derived by Fisher’s linear discriminant analysis with leave-one-out cross-validation in a TCGA-SARC training cohort, was applied without refitting to an independent external cohort (GSE36982).

**Results:**

The five-miRNA index (”Index 5”) achieved an AUC of 0.93 and, applied without refitting to an independent external cohort (GSE36982), an AUC of 0.97. After Benjamini–Hochberg correction, one candidate miRNA (miR-1305) remained significantly associated with overall survival.

**Discussion:**

These findings identify a computationally derived, externally tested candidate miRNA signature associated with sarcoma. While this study lacks experimental and clinical validation, the broader aim of this study was to come up with a framework that would assist and speedup the exploratory candidate biomarker discovery process in rare malignancies.

## 1 Introduction

Sarcomas are rare malignancies of mesenchymal origin comprising more than 70 histological subtypes that arise in bone, soft tissue, and connective structures (Sbaraglia et al., 2021). Despite their diversity, they share a common developmental lineage that fundamentally distinguishes them from other tumour classes. Whether this distinction is reflected in microRNA (miRNA) regulatory architecture—and whether such encoding carries biological and clinical significance—remains unclear. Although the mesenchymal origin of sarcoma is well established histologically, it is poorly characterised at the level of molecular regulatory programmes. In particular, it is not known whether the molecular differences separating sarcomas from other major cancer lineages represent superficial variation or deeper lineage-linked programmes associated with cellular identity, development, and tumour behaviour. Addressing this shifts the perspective from intra-sarcoma classification to the positioning of sarcoma within the broader landscape of human malignancies, with potential implications for understanding disease biology and improving clinical stratification. This question is especially relevant given the marked heterogeneity of sarcomas and the limited availability of targeted therapies and robust molecular prognostic markers (Katz et al., 2018; Wallander et al., 2023).

MicroRNAs (miRNAs) are small non-coding RNAs that regulate gene expression post-transcriptionally and retain stable tissue-of-origin signatures through malignant transformation (Bartel, 2009), (Lu et al., 2005). Large-scale studies have demonstrated that miRNA expression profiles classify tumour origin more accurately than mRNA-based approaches (Bartel, 2009), (Rosenfeld et al., 2008), indicating that lineage-specific regulatory programmes persist in cancer. These patterns primarily reflect developmental history rather than disease state, effectively encoding cellular identity. However, a key interpretive challenge is that not all miRNA differences between cancers carry equivalent biological meaning. Some represent retained lineage identity, while others reflect active oncogenic dysregulation superimposed on that identity. Distinguishing between these categories is critical, as they have fundamentally different implications for biological interpretation and clinical application.

Previous studies of sarcoma miRNAs have largely focused on tumour-versus-normal comparisons or subtype classification within sarcoma (Zoroddu et al., 2024), (Lim and Yang, 2016). These approaches do not address how sarcoma relates to other major cancer lineages at the level of regulatory architecture. To address this, we adopt a multi-lineage, one-class anomaly detection framework that systematically interrogates how sarcoma differs from epithelial, haematological, and neurological cancers. Models are trained exclusively on non-sarcoma lineages, treating sarcoma as a strictly held-out class, allowing identification of miRNAs that consistently drive sarcoma outside the expression landscape of each reference group. This design directly tests whether lineage identity is encoded in miRNA programmes: if so, models trained on developmentally distant lineages should show stronger discrimination of sarcoma than those trained on closer lineages.

Candidate miRNAs identified through this framework were subsequently evaluated using Fisher’s linear discriminant analysis (LDA) with leave-one-out cross-validation (LOOCV), which identified a compact five-miRNA panel (Index 5) within the training cohort. This frozen formula was then applied, without refitting, to an independent validation cohort, supporting the relevance of these miRNAs beyond the discovery data. Candidate microRNAs were further evaluated against normal tissue to distinguish between retained identity markers and dysregulation relative to the cell of origin. Dysregulated candidates are further assessed for prognostic relevance and functional impact through survival analysis and pathway enrichment. Through this approach, the study seeks to determine whether miRNA differences between sarcoma and other cancer lineages encode biologically interpretable programmes and clinically relevant signals. Specifically, it tests whether these differences reflect (1) retained mesenchymal lineage identity, (2) oncogenic dysregulation relative to the cell of origin, and (3) clinically meaningful phenotypes, including associations with patient survival and functional pathway activity.

## 2 Methods

### 2.1 Study design

This study employs a multi-cohort anomaly detection framework to investigate whether the microRNA (miRNA) expression landscape of sarcoma is systematically distinguishable from that of three broad cancer lineages—epithelial, haematological, and neurological. By training one-class classifiers exclusively on non-sarcoma cancer data, we operationalised sarcoma as the anomaly class, thereby asking which miRNAs are responsible for driving sarcoma samples outside the expression envelope of each reference lineage. Candidate miRNAs emerging from this discovery phase were subsequently validated through differential expression analysis against normal reference tissue and interrogated for prognostic relevance through Kaplan-Meier overall survival analysis.

### 2.2 Data sources, cohort composition and data preprocessing

MiRNA expression data were sourced from The Cancer Genome Atlas (TCGA) programme and Gene expression omnibus (GEO). A total of seven TCGA study cohorts were assembled and organised into four biological lineage groups. To reduce further bias, only primary tumor from each cohort was selected for downstream processing. The sarcoma cohort (TCGA-SARC; n = 259) comprised the held-out anomaly class and was never used during model training or feature scaling at any stage of the analysis. TCGA-SARC contains the following major subtypes–leiomyosarcoma (38.9%), liposarcoma (22.5%), and myxofibrosarcoma (9.5%), along with a mix of other smaller cohorts of sarcoma subtypes. Table 1 contains the clinical and pathological characteristics of the TCGA-SARC cohort. The epithelial lineage group (n = 2,610 total) consisted of breast carcinoma (TCGA-BRCA; n = 1,045), lung carcinoma (TCGA-LUSC and TCGA-LUAD combined; n = 956), and colorectal carcinoma (TCGA-COAD and TCGA-READ combined; n = 609). The haematological lineage group (n = 751 total) included leukaemia (TCGA-LAML, TCGA-AML, and TCGA-ALL-P2 combined; n = 563) and lymphoma (CGCI-BLGSP; n = 188). The neurological lineage group was represented by low-grade glioma (TCGA-LGG; n = 530). The microRNA expression count data was obtained from Xenabrowser (https://xenabrowser.net), which were already normalized by log2 (RPM+1) (UCSC Xena, 2026). Table 2 contains the TCGA ID and number of samples from each cohort. For differential expression validation, normal mesenchymal tissue microRNA expression profiles were obtained from The Cancer Genome Atlas (TCGA) and the Gene Expression Omnibus (GEO; https://www.ncbi.nlm.nih.gov/geo) (n = 82) (National Center for Biotechnology Information, 2002). A cross-platform normalization was carried out for all the normal samples and the two sarcoma sets so eradicate the platform bias (See Sec 6). Supplementary Figure S3 provide PCA plot of normal samples before and after normalisation. Because the TCGA-SARC cohort comprises multiple sarcoma subtypes, normal reference tissues were selected according to the tissue of origin for the predominant histological subtypes. Accordingly, normal myometrial tissue was used as the reference for leiomyosarcoma, healthy adipose tissue and adipocytes for liposarcoma, and normal fibroblasts for myxofibrosarcoma. Table 3 summarises the origin of the data for normal cohort along with the rationale for choosing. These normal samples were treated as a wholly independent reference group and were never incorporated into any training or anomaly detection step. For the validation step, only tissue samples from GSE36982 (N = 76) were used, comprising a mix of leiomyosarcoma, myxoid fibrosarcoma, liposarcoma, and a smaller cohort of other subtypes (Note: No cell line sample were chosen from GSE36982; normal sample from GSE36982 were only used for normalization; they were excluded during validation to prevent data leakage).

**TABLE 1:** Clinical and pathological characteristics of the TCGA-SARC cohort: Summary of demographic, clinical, and histopathological features of patients included in the TCGA sarcoma cohort (n = 259). Variables include age at diagnosis, sex, race, ethnicity, vital status, follow-up duration, histological categories and subtypes, primary tumour site, prior malignancy status, and treatment modalities. Continuous variables are presented as median (range), and categorical variables as counts with percentages.

Characteristic	All patients (N = 259)
Age at diagnosis (years)	
Median (range)	61 (20–90)
Sex	
Female	142 (54.2%)
Male	120 (45.8%)
Race	
White	229 (87.4%)
Black or African American	18 (6.9%)
Asian	6 (2.3%)
Not reported	9 (3.4%)
Ethnicity	
Not Hispanic or Latino	224 (85.5%)
Hispanic or Latino	5 (1.9%)
Not reported	33 (12.6%)
Vital status	
Alive	163 (62.2%)
Dead	99 (37.8%)
Follow-up/Overall survival	
Median follow-up (months, range)	31.0 (0.0–188.0)
Histological category (disease type)	
Myomatous neoplasms	105 (40.1%)
Lipomatous neoplasms	62 (23.7%)
Fibromatous neoplasms	40 (15.3%)
Soft tissue tumors and sarcomas, NOS	36 (13.7%)
Synovial-like neoplasms	10 (3.8%)
Nerve sheath tumors	9 (3.4%)
Primary diagnosis (histological subtype)	
Leiomyosarcoma, NOS	102 (38.9%)
Dedifferentiated liposarcoma	59 (22.5%)
Undifferentiated sarcoma	34 (13.0%)
Myxofibrosarcoma	25 (9.5%)
Malignant fibrous histiocytoma	12 (4.6%)
Malignant peripheral nerve sheath tumor	9 (3.4%)
Synovial sarcoma, spindle cell	6 (2.3%)
Myxoid leiomyosarcoma	3 (1.1%)
Giant cell sarcoma	3 (1.1%)
Synovial sarcoma, NOS	2 (0.8%)
Synovial sarcoma, biphasic	2 (0.8%)
Pleomorphic liposarcoma	2 (0.8%)
Other (≤1 case each)^a^	3 (1.1%)
Primary site	
Soft tissue/connective tissue	117 (44.7%)
Retroperitoneum/peritoneum	102 (38.9%)
Uterus/corpus uteri	31 (11.8%)
Other	12 (4.6%)
Prior malignancy	
Yes	42 (16.0%)
No	220 (84.0%)
Treatment received	
Chemotherapy only	47 (17.9%)
Radiation only	48 (18.3%)
Both chemotherapy and radiation	40 (15.3%)
Neither/not reported	127 (48.5%)

**TABLE 2:** Composition of TCGA cohorts grouped by cancer lineage. Overview of the study cohorts used in the analysis, organised into four lineage groups: sarcoma, epithelial, haematological, and neurological. For each group, constituent cancer types, TCGA study identifiers, and sample sizes (n) are provided. Lineage totals represent the combined number of samples within each category.

Category	Sarcoma	Epithelial			Haematological		Neurological
Sub-type	—	Breast	Lung	Colorectal	Leukaemia	Lymphoma	Glioma
TCGA study ID(s)	SARC	BRCA	LUSC, LUAD	COAD, READ	LAML, AML, ALL-P2	BLGSP	LGG
n (samples)	259	1,045	956	609	563	188	530
Lineage total	259	2,610			751		530

**TABLE 3:** Normal sample cohort.

S.No	Accession No/ID	For which subtypes	Platfom	n	Rational
1	TCGA-UCEC	Leiomyosarcoma	Illumina HiSeq	33	Normal myometrial tissue
2	GSE45363	Liposarcoma	Exiqon LNA probes version 10 microRNA array	9	Healthy adipocyte
3	GSE25470	Liposarcoma	Affymetrix multispecies miRNA-1 Array	26	Adipose tissue
4	GSE84971	Myxofibrosarcoma	nCounter human miRNA expression assay, V2	3	Fibroblasts
5	GSE18470	Liposarcoma	Agilent-019118 human miRNA microarray 2.0 G4470B	5	Adipose tissue
6	GSE159959	Leiomyosarcoma	Exiqon miRCURY LNA microRNA Array, 7th generation - hsa, mmu and rno (miRBase v18.0)	6	Normal myometrial tissue
​	TOTAL			82	​

### 2.3 Intra-lineage cohort homogeneity assessment by principal component analysis

Because the epithelial and haematological lineage groups each aggregated samples from multiple distinct TCGA study cohorts, principal component analysis (PCA) was performed prior to model training to assess whether the constituent sub-cohorts occupied a shared or segregated region of miRNA expression space. This step was motivated by the concern that a single lineage group might harbour substantial inter-cohort heterogeneity that could confound the anomaly detection task. For each lineage group containing multiple sub-cohorts, miRNA features with zero standard deviation across all samples were identified and removed prior to PCA to prevent undefined scaled values. PCA was performed on the filtered, feature-scaled matrix, applying unit-variance scaling per feature. The percentage of total variance explained by PC1 and PC2 was annotated on the respective axes. The result to this has not been included in the main text and has been presented in Supplementary Figures S1,S2.

### 2.4 One-class anomaly detection framework

#### 2.4.1 Rationale and model architecture

Three independent Isolation Forest (IF) models were trained, one per reference lineage (epithelial, haematological, and neurological) (scikit-Learn Developers, 2025). The Isolation Forest algorithm was selected for its native one-class formulation: it learns the structure of the normal class by recursively partitioning the feature space with random axis-aligned splits, assigning anomaly scores based on the mean path length required to isolate each sample across an ensemble of decision trees (scikit-Learn, 2026). Shorter mean path lengths indicate greater isolability and thus a higher anomaly score. This framework is particularly well-suited to our experimental design, in which sarcoma samples must be classified as anomalous relative to a non-sarcoma cancer reference without any sarcoma labels entering the training procedure.

Each IF model was trained exclusively on the corresponding reference lineage and subsequently evaluated on held-out validation samples from the same lineage (inliers, true negatives) and on the full sarcoma cohort (outliers, true positives). Sarcoma data were never passed to the model fitting procedure, the variance filter, or the StandardScaler at any stage, constituting a strict held-out anomaly class throughout the entire pipeline. We used empirically chosen hyperparameters for the Isolation Forest models, as follows: max-samples equals “zero-point-five,” max-features equals “zero-point-five,” contamination equals “auto,” for all the sets and seven hundred, four hundred, and two hundred trees for epithelial, haematological and neurological, respectively.

#### 2.4.2 Cross-validation strategy

Model performance was estimated using five-fold stratified ShuffleSplit cross-validation applied to the reference lineage data (train fraction: 70%; test fraction: 30%; random state: 42). At each fold, the variance filter and StandardScaler were re-fitted on the training split only, and the Isolation Forest model was re-trained from scratch with a fold-offset random seed (base seed + fold index) to ensure that tree randomness was reproducible yet non-identical across folds. Within each cross-validation fold, variance filter was applied exclusively to the training split of the reference lineage in order to prevent any information from validation or sarcoma samples influencing feature selection. Feature scaling was performed using zero-mean unit-variance standardisation (StandardScaler) fitted on the training split only; validation-split and sarcoma samples were transformed using the training-derived mean and standard deviation, ensuring that the sarcoma cohort contributed no information to the scaling parameters at any point. Sarcoma samples were scaled using the fold-specific scaler and scored against the fold-specific model at each iteration, yielding five independent anomaly score distributions.

#### 2.4.3 Decision threshold optimisation

Raw anomaly scores from Isolation Forest’s decision_function were used as continuous ranking variables for threshold-free metrics. For threshold-dependent evaluation, a constrained optimisation strategy was applied: the operating threshold was defined as the value that maximised specificity (the fraction of sarcoma samples correctly identified as anomalous) subject to the hard constraint that sensitivity (the fraction of reference lineage validation samples correctly accepted as normal) was at least 0.80. This approach assumes that maintaining a high acceptance rate for the reference lineage is the primary objective, thereby reducing false-positive anomaly calls in normal samples before optimising sarcoma detection. Accordingly, specificity was maximised only among thresholds satisfying this sensitivity constraint. In the rare event that no threshold achieved the minimum sensitivity requirement, the Youden index point (maximising J = sensitivity + specificity −1) was used as a fallback, and such folds were flagged in the results (Ruopp et al., 2008).

#### 2.4.4 Performance evaluation

Threshold-free discrimination was quantified using the area under the receiver operating characteristic curve (AUC-ROC) and the area under the precision-recall curve (AUC-PR, equivalent to average precision). Threshold-dependent performance at the constrained operating point was reported as sensitivity, specificity, and F1 score. All metrics were computed separately for each fold and summarised as mean ± standard deviation across the five folds. ROC and precision-recall curves were visualised with per-fold individual curves overlaid on the mean curve ± one standard deviation band.

### 2.5 SHAP-based feature importance

Feature importance was computed using exact TreeSHAP (SHapley Additive exPlanations) (Yang, 2022), (SHAP Developers, 2026). This perturbation strategy uses the conditional distribution implied by the tree structure to account for the correlation among miRNA expression features, which is the theoretically appropriate choice for high-dimensional, collinear biological data. In contrast to marginal SHAP approaches that require a background dataset, tree-path-dependent TreeSHAP reads tree structure directly and requires no background sampling, eliminating any risk of inadvertent sarcoma information leaking into the importance computation.

At each cross-validation fold, SHAP values were computed for all sarcoma samples using the fold-specific fitted Isolation Forest model and the fold-specific scaled sarcoma expression matrix. The Isolation Forest decision function assigns lower (more negative) scores to outlier samples; consequently, a feature with a negative mean SHAP value pulls the decision score downward and therefore acts as an outlier driver. To produce an importance metric with a positive orientation (higher value = stronger outlier driver), raw mean SHAP values were negated prior to accumulation. The resulting per-fold importance vectors were averaged across all five folds to yield a single fold-averaged SHAP importance score per miRNA per cohort comparison. MiRNAs were subsequently ranked in descending order of this fold-averaged importance, and the top 30 miRNAs from each lineage comparison were retained as candidate sarcoma anomaly-associated features.

To identify miRNAs whose anomaly-driving signal was lineage-independent, a cross-cohort overlap analysis was performed on the top-30 feature lists derived from each of the three Isolation Forest comparisons. Supplementary Table S1 contains the full list of 30 miRNAs for each cohort along with their SHAP score. A binary presence matrix was constructed indicating whether each miRNA appeared in the top-30 list of each lineage comparison. MiRNAs present in the top-30 lists of two or more lineage comparisons were classified as shared candidates; those present across all three comparisons were designated universal markers. Only those microRNAs indicated in SHAP analysis as important (present in two or more comparison) was taken forward to subsequent stages.

### 2.6 Cross-platform normalization

Before validation and differential analysis, miRNA expression profiles from RNA-sequencing and microarray datasets were harmonized via platform-specific normalization. Datasets on a linear scale (95th percentile expression >50) were log2 (x+1) transformed; datasets already log-scaled were left unchanged. miRNA-wise z-score normalization was then applied, with baseline mean and SD derived from each dataset’s own matched normal reference samples. However, our code had a fallback feature that if there are fewer than two samples in any set, it will borrow the SD from the largest group. This was not used in our study but was still kept in the codebase to help future research with very low sample numbers. Supplementary Table S2 summarises the scale of the data, the platform on which they were processed and for which datasets log2 transformation was applied.

### 2.7 Validation of SHAP identified miRNAs using Fisher’s LDA and LOOCV

To validate the relevance of the miRNAs identified through SHAP analysis, a Fisher linear discriminant analysis (LDA)-based miRNA index was constructed following the strategy of Asano et al. (2019). The TCGA sarcoma cohort was used as the training sarcoma cohort, while the independent GSE36982 cohort was reserved as an external validation sarcoma cohort. The pooled normal samples were stratified by accession and proportionally divided into training and validation subsets according to the relative size of the TCGA-SARC and GSE36982 sarcoma cohorts. (Note: No cell line sample were chosen from GSE36982; normal sample from GSE36982 were only used for normalization; they were excluded during validation to prevent data leakage). The selected candidate miRNAs were used as the input feature pool, and all possible combinations of miRNAs were systematically evaluated using Fisher’s LDA with leave-one-out cross-validation (LOOCV) in the training dataset (TCGA sarcoma cohort combined with the training subset of normal samples). LDA models were trained using equal class priors (0.5 probability for sarcoma and normal classes) to avoid bias caused by class imbalance. For each index size, the combination achieving the highest LOOCV AUC was retained, allowing assessment of whether a reduced marker panel could achieve comparable performance to the complete candidate set.

The optimal index (The miRNA index represents a weighted linear combination of selected miRNA expression levels, where each miRNA contributes to the final classification score according to its LDA-derived coefficient. Index 2 represents LDA model was formed using 2 microRNAs and Index 3 represents it was formed using 3 microRNAs and so on) was selected using a performance plateau criterion, in which additional miRNAs were excluded if they did not provide meaningful improvement in performance. The final LDA model was then refitted using the selected miRNAs on the complete training dataset, generating a fixed equation consisting of miRNA-specific coefficients and an intercept. In this study, the resulting indices included, for example, a three-miRNA model: Index 3 = (0.9365 * hsa-mir-196a) + (0.2502 * hsa-mir-767) + (0.3596 * hsa-mir-1245a) + (−1.8218), and a four-miRNA model: Index 4 = (0.8598 * hsa-mir-196a) + (0.2027 * hsa-mir-767) + (0.3119 * hsa-mir-1245a) + (0.2835 * hsa-mir-493) + (−1.9650). A fixed decision threshold (Index ≥0 classified as sarcoma and Index <0 as normal) was applied for all subsequent analyses.

External validation was performed by applying the frozen LDA formula to an independent cohort consisting of GSE36982 sarcoma samples and the held-out normal validation subset. The external dataset was not used for feature selection, index optimization, coefficient estimation, or threshold adjustment. Performance was evaluated by receiver operating characteristic (ROC) analysis and calculation of sensitivity, specificity, accuracy, balanced accuracy, and AUC, providing an unbiased assessment of the generalizability of the SHAP/Isolation Forest-derived miRNA candidates.

### 2.8 Differential expression analysis against normal tissue and functional analysis

To contextualise the anomaly-driving miRNA signatures within a biological frame of reference, differential expression analysis was performed comparing TCGA sarcoma miRNA profiles against matched and pooled normal mesenchymal tissue (Table 3).

For each miRNA, a two-sided Mann-Whitney U test was performed (Chicco et al., 2025). This non-parametric test was selected in preference to a t-test because of the highly unequal sample sizes between groups and because it does not require the assumptions of normally distributed data or equal variances. The test statistic U was also used to compute the rank-biserial correlation coefficient as an effect size measure: r = 2U/(n_1_ × n_2_) − 1, which ranges from −1 (all normal samples exceeding all sarcoma samples) to +1 (all sarcoma samples exceeding all normal samples), providing a standardised and interpretable measure of the magnitude and direction of the expression difference independent of sample size. As the expression data had been pre-normalized to z-scores, differential expression was quantified as the difference in median normalized expression between the sarcoma and normal groups (Sarcoma − Normal). Multiple testing correction was performed using the Benjamini–Hochberg (BH) procedure to control the false discovery rate (FDR) (Chen et al., 2017). MiRNAs were considered significantly differentially expressed if they satisfied both an FDR-adjusted p-value of less than 0.05 and an absolute median expression difference of at least 0.5 z-score units.

To explore the downstream biological consequences of the consensus sarcoma-associated miRNA signature, miRNA–gene interaction network construction and pathway enrichment analysis were performed using the web-based platform MiENTURNet (http://userver.bio.uniroma1.it/apps/mienturnet/) (Licursi et al., 2019). MiENTURNet was used to retrieve experimentally validated and high-confidence predicted miRNA–target gene interactions, from which an interaction network was constructed with miRNAs and their respective target genes represented as nodes and regulatory relationships as directed edges. Network topology was examined to identify hub genes—target genes subject to regulation by multiple consensus miRNAs—as these represent convergence points likely to be of functional relevance in sarcoma biology. To increase the biological interpretability of the network plot, only those interactions important for sarcoma were retained. Pathway enrichment analysis of the network target gene set was subsequently performed against the KEGG and Reactome pathway databases, as implemented within the MiENTURNet platform. The enrichment results were interpreted in the context of known sarcoma biology, with particular attention to pathways pertaining to mesenchymal cell identity, cell cycle regulation, extracellular matrix remodelling, and oncogenic signalling cascades.

### 2.9 Kaplan-Meier survival analysis

To assess the prognostic relevance of the candidate miRNAs identified through the anomaly detection and differential expression analyses, Kaplan-Meier (KM) overall survival analysis was performed within the sarcoma patient cohort. For each candidate miRNA, the sarcoma patients were dichotomised into high-expression and low-expression groups based on the median miRNA expression value across the cohort: patients with expression values strictly above the median were assigned to the high group, and those at or below the median were assigned to the low group.

Kaplan-Meier survival curves were estimated independently for the high and low expression groups using the KaplanMeierFitter class from the lifelines library. The primary survival endpoint was overall survival, defined as the time from initial diagnosis (or equivalent clinical reference point) to death from any cause (event indicator: OS = 1) or last follow-up (censoring indicator: OS = 0), with time reported in months. Statistical comparison between the high and low expression survival curves was performed using the log-rank test, which assesses the null hypothesis of no difference in survival distributions between the two groups across all observed time points. For each miRNA, the median overall survival time was extracted for both the high and low expression groups; miRNAs for which the survival curve did not reach 50% probability within the observation window were reported as “not reached.” Individual KM plots were generated for each candidate miRNA, displaying the estimated survival functions for both expression groups with 95% confidence bands, the log-rank p-value annotated within the figure panel, and group sample sizes reported in the legend.

### 2.10 Software and statistical computing environment

All anomaly detection, SHAP, differential expression, and survival analyses were implemented in Python 3.14.3. Core scientific computing libraries used included NumPy, pandas, scikit-learn (Isolation Forest, StandardScaler, ShuffleSplit, performance metrics), SHAP (TreeExplainer), SciPy (Mann-Whitney U test), statsmodels (Benjamini–Hochberg FDR correction), and lifelines (Kaplan-Meier estimation and log-rank testing). Data visualisation was performed using matplotlib and seaborn. PCA and associated visualisations were performed in R (version 4.5.0) using the tidyverse, reshape2, and ggplot2 packages. A global random seed of 42 was applied across all stochastic procedures to ensure full reproducibility.

## 3 Results

Three independent Isolation Forest models were trained on epithelial, haematological, and neurological cancer miRNA expression profiles and evaluated using a strictly held-out sarcoma cohort across five cross-validation folds. Overall performance, including ROC curves, precision–recall curves, and operating characteristics, consistently demonstrated that sarcoma miRNA profiles are separable from each reference lineage. All comparisons achieved AUC-ROC values well above chance, with the neurological model showing the strongest discrimination (0.963 ± 0.010), followed by the haematological (0.891 ± 0.014) and epithelial (0.858 ± 0.015) models. Fold-to-fold variability remained low, indicating stable and reproducible performance.

Precision–recall analysis reinforced these findings, with high mean average precision observed across all models: 0.965 ± 0.009 for neurological, 0.921 ± 0.009 for epithelial, and 0.955 ± 0.005 for haematological comparisons. Notably, the epithelial model exhibited higher average precision than AUC-ROC, suggesting strong precision at moderate recall despite the greater heterogeneity and complexity of the epithelial dataset. Across folds, metric distributions showed that the neurological model achieved near-perfect specificity (0.998 ± 0.004), while haematological (0.917 ± 0.082) and epithelial (0.724 ± 0.049) models showed progressively lower specificity. Importantly, the predefined sensitivity constraint (≥0.80) was consistently satisfied across all folds without fallback adjustments, with tightly bounded sensitivity values for epithelial (0.802 ± 0.001), haematological (0.803 ± 0.004), and neurological (0.850 ± 0.021) models.

Analysis of operating points revealed a clear gradient in performance, with the neurological model combining high sensitivity and near-perfect specificity, while epithelial and haematological models showed progressively reduced specificity. Threshold analysis further demonstrated strong stability across folds, with low variance in optimal thresholds for each cohort (epithelial: 0.135 ± 0.002; haematological: 0.112 ± 0.008; neurological: 0.098 ± 0.005), indicating consistent anomaly score distributions and robustness to data partitioning. Figure 1 and Table 4 shows the data for performance of Isolation Forest Anomaly Detection.

**FIGURE 1:**
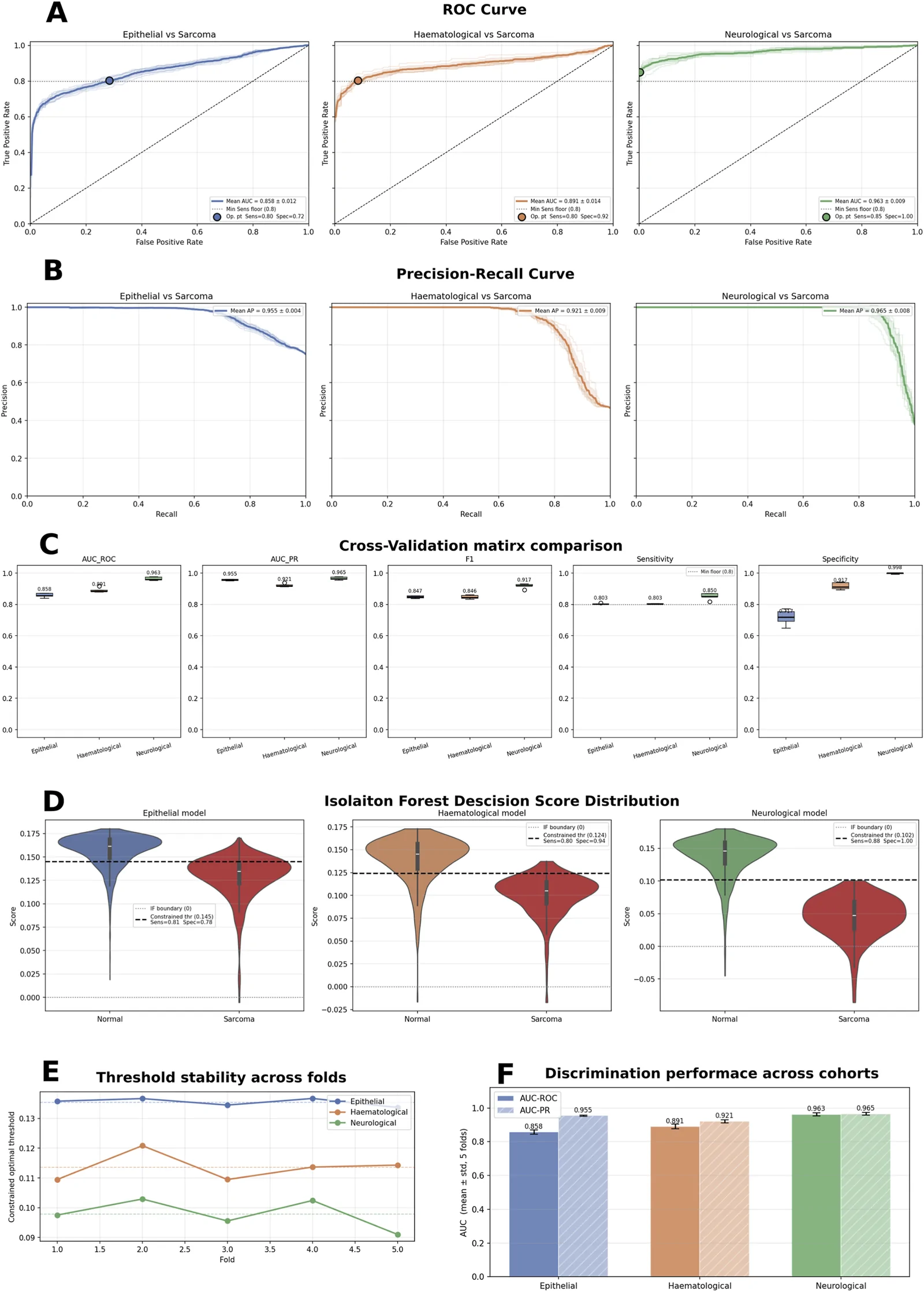
Isolation Forest anomaly detection performance across cancer lineages. **(A)** Receiver operating characteristic (ROC) curves for epithelial, haematological, and neurological models across five cross-validation folds, with mean curve ±1 SD and constrained operating points indicated. **(B)** Precision–recall curves with mean average precision (AUC-PR) shown for each lineage. **(C)** Box plots summarising fold-wise distributions of AUC-ROC, AUC-PR, F1-score, sensitivity, and specificity. **(D)** Decision Score distribution for each comparison. **(E)** Stability of decision thresholds across folds for each lineage model. **(F)** Summary comparison of AUC-ROC and AUC-PR across the three lineage-specific models.

**TABLE 4:** Performance metrics of Isolation Forest anomaly detecjtion models. Summary of model performance across five cross-validation folds for each lineage-specific Isolation Forest model (epithelial, haematological, neurological). Metrics include area under the receiver operating characteristic curve (AUC-ROC), area under the precision–recall curve (AUC-PR), F1-score, sensitivity, specificity, and optimised decision threshold. Values are reported as mean ± standard deviation.

Cohort	AUC-ROC	AUC-PR	F1-score	Sensitivity	Specificity	Threshold
Epithelial	0.8585 ± 0.0145	0.9549 ± 0.0047	0.8473 ± 0.0074	0.8020 ± 0.0009	0.7243 ± 0.0493	0.1354 ± 0.0018
Haematological	0.8910 ± 0.014	0.9210 ± 0.009	0.8460 ± 0.0399	0.8030 ± 0.0037	0.917 ± 0.0821	0.1117 ± 0.0079
Neurological	0.9632 ± 0.0104	0.9651 ± 0.0090	0.9171 ± 0.0141	0.8503 ± 0.0206	0.9977 ± 0.0035	0.0979 ± 0.0050

TreeSHAP-based feature importance analysis, applied fold-by-fold to sarcoma samples scored against each lineage-specific Isolation Forest model, yielded ranked lists of the top 30 anomaly-driving miRNAs per comparison. Cross-lineage overlap analysis of these ranked lists identified eight consensus candidate miRNAs that appeared in the top-30 lists of two or more lineage comparisons (Table 5). hsa-miR-337 was the sole miRNA present across all three lineage comparisons—epithelial, haematological, and neurological. The remaining miRNAs (hsa-miR-196a, hsa-miR-214, hsa-miR-1305, hsa-miR-1245a, hsa-miR-767, hsa-miR-493, and hsa-miR-3152) each appeared in exactly two of the three comparisons. Among these, hsa-miR-196a carried the highest mean fold-averaged SHAP importance score (0.030), followed by hsa-miR-214 (0.026) and hsa-miR-1305 (0.021), indicating that these three miRNAs were the most consistent drivers of the sarcoma anomaly score within their respective comparison pairs. These eight miRNAs were taken forward as the consensus sarcoma-associated miRNA signature for all downstream validation analyses.

**TABLE 5:** Consensus miRNAs identified by SHAP-based feature importance. List of miRNAs consistently identified as anomaly-driving features across lineage comparisons based on fold-averaged TreeSHAP importance scores. The table reports each miRNA identifier, its mean SHAP importance score, and the number of lineage comparisons (n_cohorts) in which it appeared among the top-ranked features.

MicroRNA ID	SHAP importance score	N cohorts
hsa-miR-196a	0.030	2
hsa-miR-767	0.017	2
hsa-miR-214	0.026	2
hsa-miR-1305	0.021	2
hsa-miR-1245a	0.017	2
hsa-miR-493	0.016	2
hsa-miR-337	0.015	3
hsa-miR-3152	0.015	2

MiR-337 known target repertoire is remarkably restricted; MiENTURNet confirmed only a single validated target gene, HOXB7, which precludes its inclusion in multi-target pathway enrichment analyses but does not diminish its biological candidacy.

### 3.1 Validation of SHAP-identified miRNAs using Fisher’s LDA and LOOCV

Validation index for sarcoma was derived through a combinatorial Fisher’s LDA with LOOCV. Out of the eight microRNAs identified by SHAP analysis, hsa-miR-3152 was eliminated from validation and differential expression analysis since this microRNA species was annotated in very few GEO samples but was used in subsequent downstream analysis. Each of the remaining seven candidate microRNAs (hsa-miR-196a, hsa-miR-214, hsa-miR-767, hsa-miR-1245a, hsa-miR-1305, hsa-miR-337 and hsa-miR-493) was first assessed individually within the training cohort. Single-marker performance was poor and variable, with hsa-miR-196a alone providing the strongest individual training-cohort discrimination (AUC = 0.78; sensitivity 0.67, specificity 0.90), while the remaining single markers achieved training AUCs of 0.56–0.67. Single-marker external validation was also highly variable: several individual miRNAs performed comparably or better in the validation cohort than in training (e.g., hsa-miR-1305, training AUC 0.64 vs. validation AUC 0.98), while others degraded substantially, most notably hsa-miR-767 (training AUC 0.65 vs. validation AUC 0.48, below chance), underscoring that no single miRNA provides reliable, generalizable discrimination on its own.

All possible combinations of the seven candidates were then exhaustively evaluated by LOOCV-LDA for panel sizes of two to seven markers, retaining the best-performing combination at each size within the training cohort only. Discriminative performance rose sharply from a single marker to two-marker panels (AUC = 0.88) and continued to improve up to five markers (AUC = 0.93), after which additional markers yielded no further gain (AUC = 0.93 for both the six- and seven-marker panels; Table 6; Figure 2). Applying a pre-specified parsimony criterion—the smallest panel size whose LOOCV AUC fell within 0.005 of the maximum AUC achieved across all panel sizes—the five-marker combination of hsa-miR-196a, hsa-miR-767, hsa-miR-1245a, hsa-miR-1305 and hsa-miR-493 was selected as the final index (“Index 5”). This panel achieved a training-cohort LOOCV AUC of 0.93, with sensitivity 0.75, specificity 1.00, accuracy 0.80 and balanced accuracy 0.87. The discriminant formula for Index 5, fit once on the full training cohort, was: Index 5 = (0.8353 * hsa-mir-196a) + (0.1943 * hsa-mir-767) + (0.2874 * hsa-mir-1245a) + (0.0282 * hsa-mir-1305) + (0.2665 * hsa-mir-493) + (−1.9598); with the classification rule Index 5 ≥ 0 indicating sarcoma and Index 5 < 0 indicating normal, consistent with the equal-prior LDA decision boundary. This formula was frozen at this point and was not modified in any subsequent analysis. Figure 3 shows the ROC-AUC curves for the training versus validation sets.

**TABLE 6:** Validation performance of individual microRNAs and combinatorial panels, training and validation cohorts. Sensitivity, specificity, accuracy, balanced accuracy, and AUC are reported for each of the seven candidate miRNAs individually and for the best-performing combination at each panel size (2–7), evaluated by leave-one-out cross-validation (LOOCV) in the training cohort and by single, non-refit application to the independent external validation cohort. The five-marker panel (Index 5) was selected via a pre-specified parsimony criterion (see Methods).

​	​	Training cohort					Validation cohort				
Index size	Markers	Sensitivity	Specificity	Accuracy	Balanced accuracy	AUC	Sensitivity	Specificity	Accuracy	Balanced accuracy	AUC
1	hsa-mir-196a	0.67	0.90	0.72	0.79	0.78	0.92	0.79	0.90	0.86	0.95
1	hsa-mir-214	0.56	0.83	0.61	0.69	0.67	0.95	0.79	0.92	0.87	0.91
1	hsa-mir-767	0.47	0.87	0.55	0.67	0.65	0.04	0.89	0.21	0.47	0.48
1	hsa-mir-1245a	0.54	0.94	0.62	0.74	0.66	0.69	1.00	0.75	0.85	0.85
1	hsa-mir-1305	0.50	1.00	0.60	0.75	0.64	0.90	0.95	0.91	0.92	0.98
1	hsa-mir-337	0.52	0.83	0.58	0.67	0.56	0.77	0.79	0.77	0.78	0.87
1	hsa-mir-493	0.57	0.89	0.63	0.73	0.63	0.56	0.84	0.62	0.70	0.75
2	hsa-mir-196a, hsa-mir-1245a	0.73	0.95	0.78	0.84	0.88	0.94	0.84	0.92	0.89	0.96
3	hsa-mir-196a, hsa-mir-767, hsa-mir-1245a	0.74	0.98	0.79	0.86	0.91	0.88	0.89	0.89	0.89	0.96
4	hsa-mir-196a, 767, 1245a, 493	0.76	1.00	0.80	0.88	0.92	0.88	0.95	0.90	0.92	0.96
5	hsa-mir-196a, 767, 1245a, 1305, 493	0.75	1.00	0.80	0.87	0.93	0.92	0.95	0.93	0.94	0.97
6	hsa-mir-196a, 767, 1245a, 1305, 337, 493	0.73	0.98	0.78	0.86	0.93	0.85	0.95	0.87	0.90	0.96
7	hsa-mir-196a, 214, 767, 1245a, 1305, 337, 493	0.73	0.98	0.78	0.86	0.93	0.83	0.95	0.86	0.89	0.96

**FIGURE 2:**
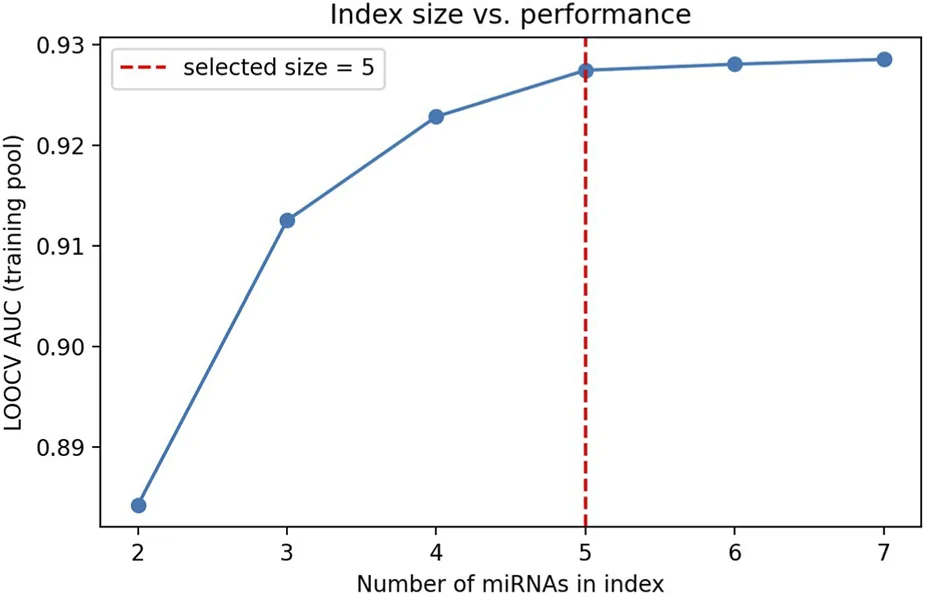
Relationship between microRNA panel size and LOOCV AUC in the training cohort. AUC increased with panel size up to markers and plateaued thereafter (dashed line showing optimal performance index).

**FIGURE 3:**
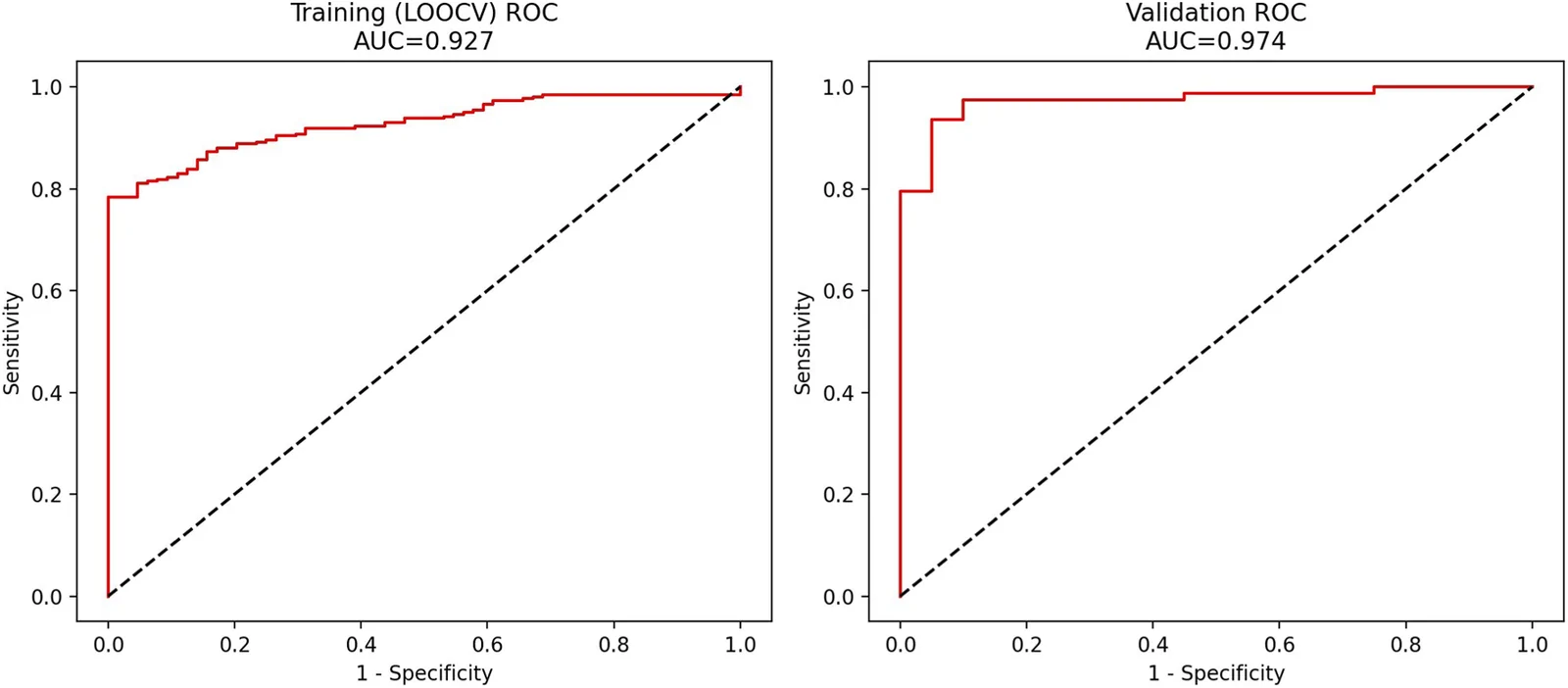
Receiver operating characteristic (ROC) curves for the Index 5 in the training cohort (left, LOOCV) and the independent GSE36982 validation cohort (right, single application of the frozen formula).

The frozen Index 5 formula, derived using only the training cohort, was applied without any refitting or adjustment to the independent GSE36982 sarcoma cohort and its paired normal-validation subset. Performance in this external, single-application test numerically exceeded the internal LOOCV estimate, with an AUC of 0.97, sensitivity of 0.92, specificity of 0.95, accuracy of 0.93 and balanced accuracy of 0.94. Table 6 reports training and validation performance side by side for every individual marker and every combinatorial panel size (2–7), following the reporting convention of Asano et al. (2019).

### 3.2 Differential expression of consensus miRNAs against normal tissue

To distinguish miRNAs that represent sarcoma-specific alterations from those identified only through cancer-to-cancer comparisons, the seven consensus miRNAs were further evaluated by differential expression analysis comparing TCGA sarcoma samples with normal tissue profiles. The results are summarised in Table 7. Following Benjamini–Hochberg (BH) correction, all seven consensus miRNAs showed statistically significant differential expression at an adjusted p-value threshold of <0.05, with all significant miRNAs exhibiting increased expression in sarcoma relative to normal tissue.

**TABLE 7:** Differential expression of consensus miRNAs in harmonized sarcoma versus normal tissue. Results of differential expression analysis comparing TCGA sarcoma samples with normal reference tissue. Metrics include the mean and median difference in z-scores ( ΔZ ), Mann–Whitney U statistic, raw p-value, Benjamini–Hochberg adjusted p-value, and rank-biserial correlation coefficient as an effect size measure.

microRNA ID	ΔZ median	ΔZ mean	U statistic	P value	Adj. p-value (BH)	Effect Size(r)
hsa-mir-196a	2.26	1.73	17,698	5.9E-18	4.1E-17	0.627
hsa-mir-767	1.51	2.12	16,441	1.8E-12	6.4E-12	0.511
hsa-mir-214	1.58	1.31	15,903	2.0E-10	4.6E-10	0.462
hsa-mir-1305	3.09	5.56	14,843	4.0E-07	5.6E-07	0.364
hsa-mir-1245a	1.59	1.75	14,920	3.1E-07	5.4E-07	0.372
hsa-mir-493	2.29	1.52	13,958	9.6E-05	1.1E-04	0.283
hsa-mir-337	1.38	0.33	12,505	3.9E-02	3.9E-02	0.150

Among the identified miRNAs, miR-196a, miR-767, and miR-214 demonstrated the strongest statistical evidence of upregulation. This was supported not only by highly significant p-values, but also by large effect sizes (*r* = 0.627, 0.511, and 0.462, respectively) and median ΔZ of 2.26, 1.51, and 1.58. MiR-1305 displayed one of the largest expression shifts, with a median ΔZ of 3.09 and a mean ΔZ of 5.56, accompanied by a highly significant adjusted p-value of 5.6E-07 and a moderate effect size (*r* = 0.364). This was followed by miR-1245a and miR-493 with median ΔZ of 1.59 and 2.29 respectively. MiR-337 showed a weaker but still statistically significant increase in expression (median ΔZ = 1.38, adj. p = 0.039), which is consistent with its very small effect size (*r* = 0.150), suggesting a more modest shift relative to normal tissue.

This finding demonstrates that most consensus miRNAs identified by the machine learning framework represent transcriptional abnormalities in sarcoma when compared with normal tissue. In particular, hsa-miR-196a, hsa-miR-767, hsa-miR-214, hsa-miR-1305, hsa-miR-1245a, and hsa-miR-493 show strong evidence of sarcoma-associated upregulation.

#### 3.2.1 miRNA–gene interaction network and pathway enrichment analysis

To translate the consensus miRNA signature into a functional biological framework, miRNA–target gene interaction network analysis was performed using MiENTURNet, retaining only those target gene interactions with established relevance to sarcoma biology. The resulting network (Figure 4) illustrates convergent regulation of sarcoma-relevant gene nodes by multiple consensus miRNAs, with hsa-miR-1305, hsa-miR-493, and hsa-miR-3152 sharing regulatory relationships with several common target genes, and hsa-miR-337 connecting exclusively to HOXB7. The hub structure of the network identifies candidate effector genes through which the consensus miRNA signature may collectively remodel the sarcoma transcriptome.

**FIGURE 4:**
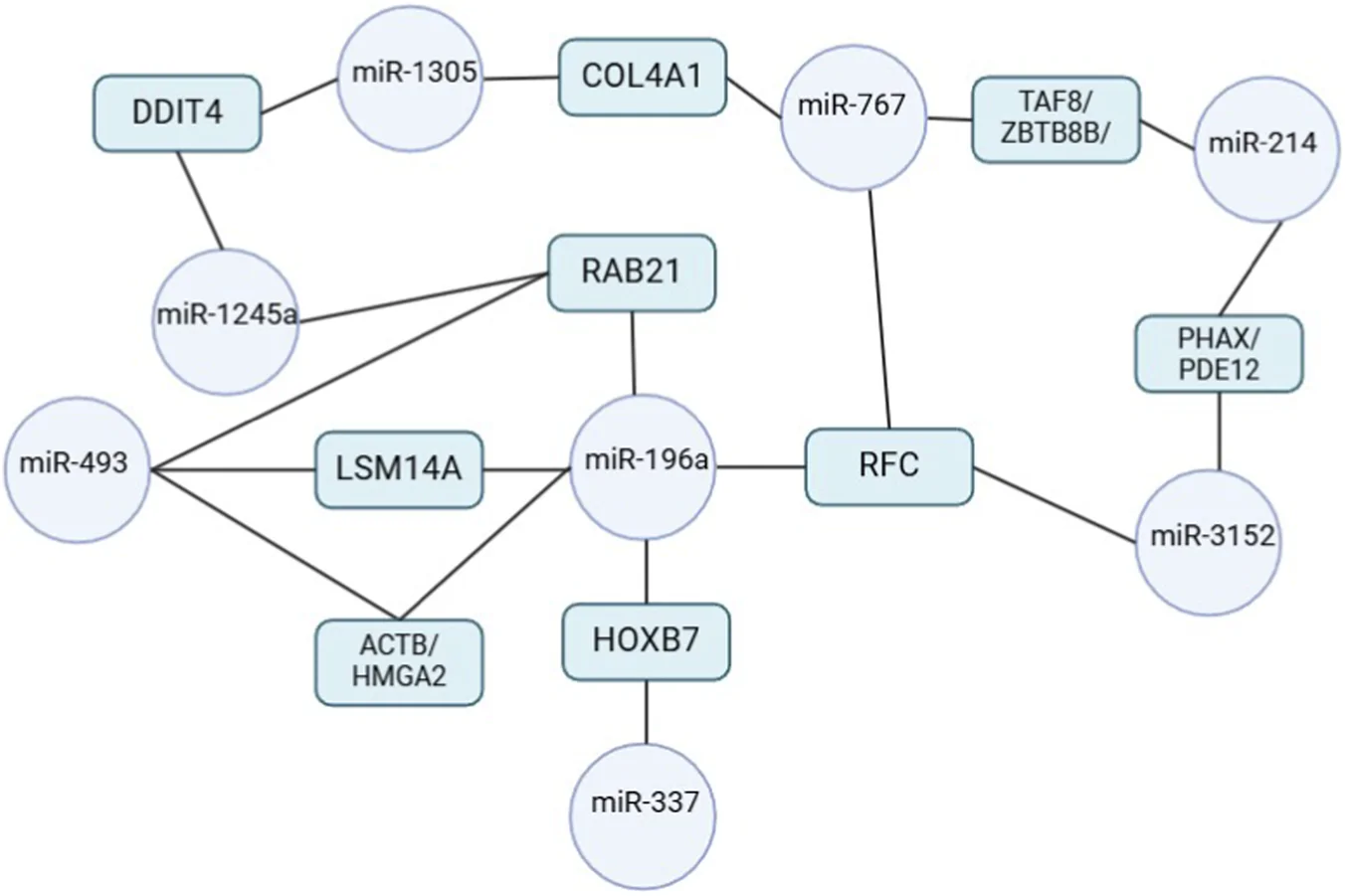
miRNA–gene interaction network of consensus sarcoma-associated miRNAs. Network representation of interactions between the seven consensus miRNAs and their experimentally validated or high-confidence predicted target genes, constructed using MiENTURNet. Nodes represent miRNAs and target genes, and edges denote regulatory interactions. Hub genes regulated by multiple miRNAs are highlighted, indicating points of convergence likely to be functionally relevant in sarcoma biology.

For pathway enrichment analysis, hsa-miR-337 was excluded, as its single validated target gene (HOXB7) does not provide sufficient statistical power for hypergeometric enrichment testing. KEGG pathway analysis of the remaining seven miRNAs’ target gene set revealed significant enrichment across multiple oncogenic and stress-response pathway categories (Figure 5). Among the most relevant to sarcoma biology were the PI3K-AKT signalling pathway, the mTOR signalling pathway, and the p53 signalling pathway. Focal adhesion and integrin signalling were also enriched, consistent with the known dependence of mesenchymal tumour cells on extracellular matrix mechanosensing for invasion and metastasis. DNA replication fidelity and repair pathways—including mismatch repair, base excision repair, nucleotide excision repair, and ATP-dependent chromatin remodelling—featured prominently, pointing to a role for this miRNA cluster in promoting the genomic instability that characterises high-grade sarcoma. Cell cycle regulation and transcriptional misregulation in cancer further reinforced a proliferative and dedifferentiation phenotype. AMPK signalling and autophagy pathways were additionally enriched, suggesting modulation of metabolic stress responses.

**FIGURE 5:**
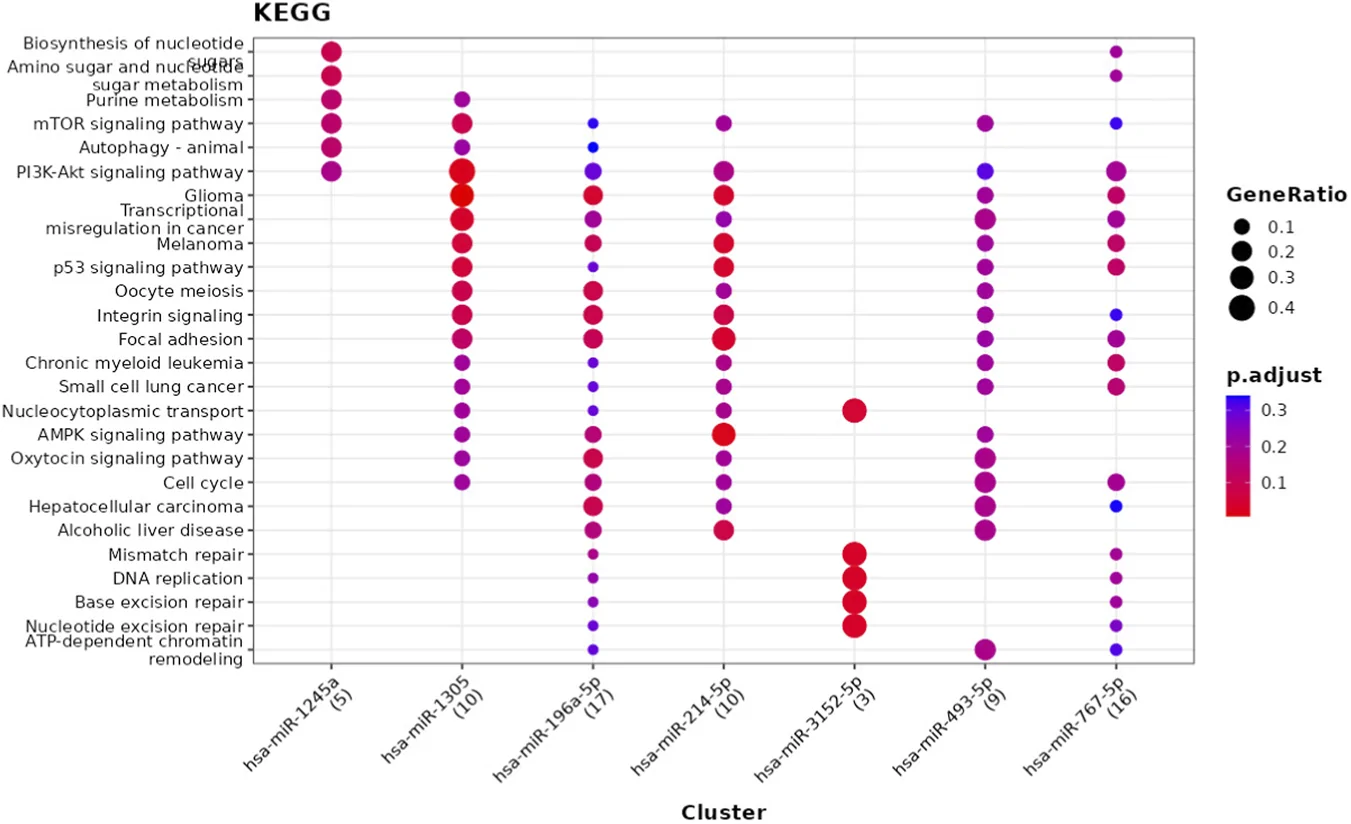
KEGG pathway enrichment analysis of consensus miRNA target genes. Dot plot showing significantly enriched KEGG pathways derived from the target gene set of the seven consensus miRNAs (excluding miR-337). Size of the dot indicates the number of genes involved in the particular pathway for a particular microRNA. Colour scale indicate the adjusted p value significance of the dot (Red being more significant than blue).

Reactome pathway analysis corroborated and extended these findings (Figure 6). Regulation of TP53 activity—including through phosphorylation and in the context of DNA damage- and telomere stress-induced senescence—was a prominent theme, converging with the KEGG p53 and DNA repair findings to implicate the consensus signature in the suppression of oncogene-induced and damage-induced senescence checkpoints. Consistent with this, Oncogene Induced Senescence and Oxidative Stress Induced Senescence were both significantly enriched, suggesting that the miRNA network may contribute to sarcoma progression by attenuating senescence surveillance mechanisms. Regulation of RUNX1 expression and activity was a particularly interesting enrichment: RUNX1, while primarily characterised in haematopoiesis, has an emerging role in mesenchymal cell fate decisions and has been implicated in sarcoma subtypes. S Phase, Chromosome Maintenance, Cohersin Loading onto Chromatin, and translesion synthesis pathways were enriched in keeping with the KEGG DNA repair findings, jointly reinforcing a picture of compromised genome surveillance. RAB geranylgeranylation and RAB-mediated vesicular trafficking pathways were also enriched, which may reflect the known role of endosomal trafficking in sustaining receptor tyrosine kinase signalling in sarcoma. Estrogen receptor-mediated and broader nuclear receptor signalling pathways also appeared in the Reactome results, a finding of potential interest in the context of hormonally responsive sarcoma subtypes.

**FIGURE 6:**
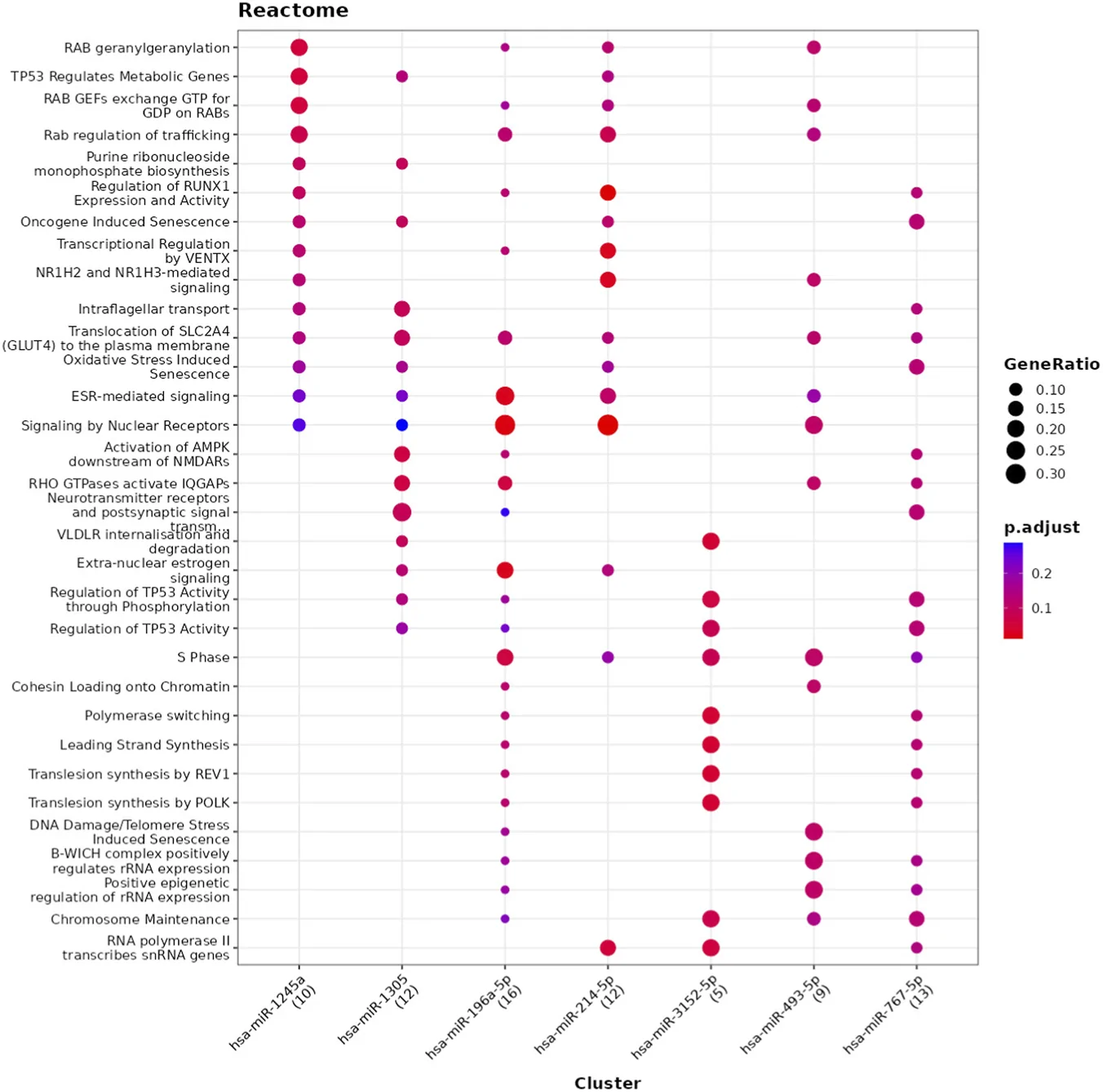
Reactome pathway enrichment analysis of consensus miRNA target genes. Dot plot of significantly enriched Reactome pathways associated with the consensus miRNA target gene set. Size of the dot indicates the number of genes involved in the particular pathway for a particular microRNA. Colour scale indicate the adjusted p value significance of the dot (Red being more significant than blue).

Collectively, the KEGG and Reactome enrichment profiles converge on four broad biological themes: (1) activation of the PI3K-AKT-mTOR oncogenic axis, (2) impairment of the p53-mediated DNA damage response and senescence programme, (3) dysregulation of cell cycle entry and genome maintenance, and (4) remodelling of the mesenchymal cytoskeletal and adhesion programme.

### 3.3 Prognostic significance of consensus miRNAs in sarcoma overall survival

Kaplan–Meier overall survival analysis was performed for all eight consensus miRNAs within the TCGA-SARC cohort. The total sample was dichotomised into high- and low-expression groups based on a median split of per-miRNA expression. Log-rank p-values were corrected for multiple testing using the Benjamini–Hochberg (BH) procedure across all eight comparisons to control the false discovery rate. Results are summarised in Table 8, and the corresponding Kaplan–Meier plots are shown in Figure 7.

**TABLE 8:** Kaplan–Meier survival analysis of consensus miRNAs in sarcoma. Summary of overall survival analysis for sarcoma patients stratified by median expression of each consensus miRNA. The table reports log-rank test p-values and median overall survival (months) for high- and low-expression groups. Significant associations indicate miRNAs with potential prognostic relevance.

microRNA ID	Log rank P-value	Adj. P-value (BH)	Median OS high (months)	Median OS low (months)
hsa-mir-196a	0.588	0.672	63.76	66.82
hsa-mir-767	0.029	0.116	53.45	84.59
hsa-mir-214	0.095	0.19	48.55	80.95
hsa-mir-1305	**0.003**	**0.024**	48.55	85.38
hsa-mir-1245a	0.075	0.19	54.17	80.95
hsa-mir-493	0.516	0.672	60.61	80.42
hsa-mir-337	0.475	0.672	60.61	80.42
hsa-mir-3152	0.858	0.858	66.82	65.41

After Benjamini–Hochberg correction, one candidate miRNA (miR-1305) remained significantly associated with overall survival.

**FIGURE 7:**
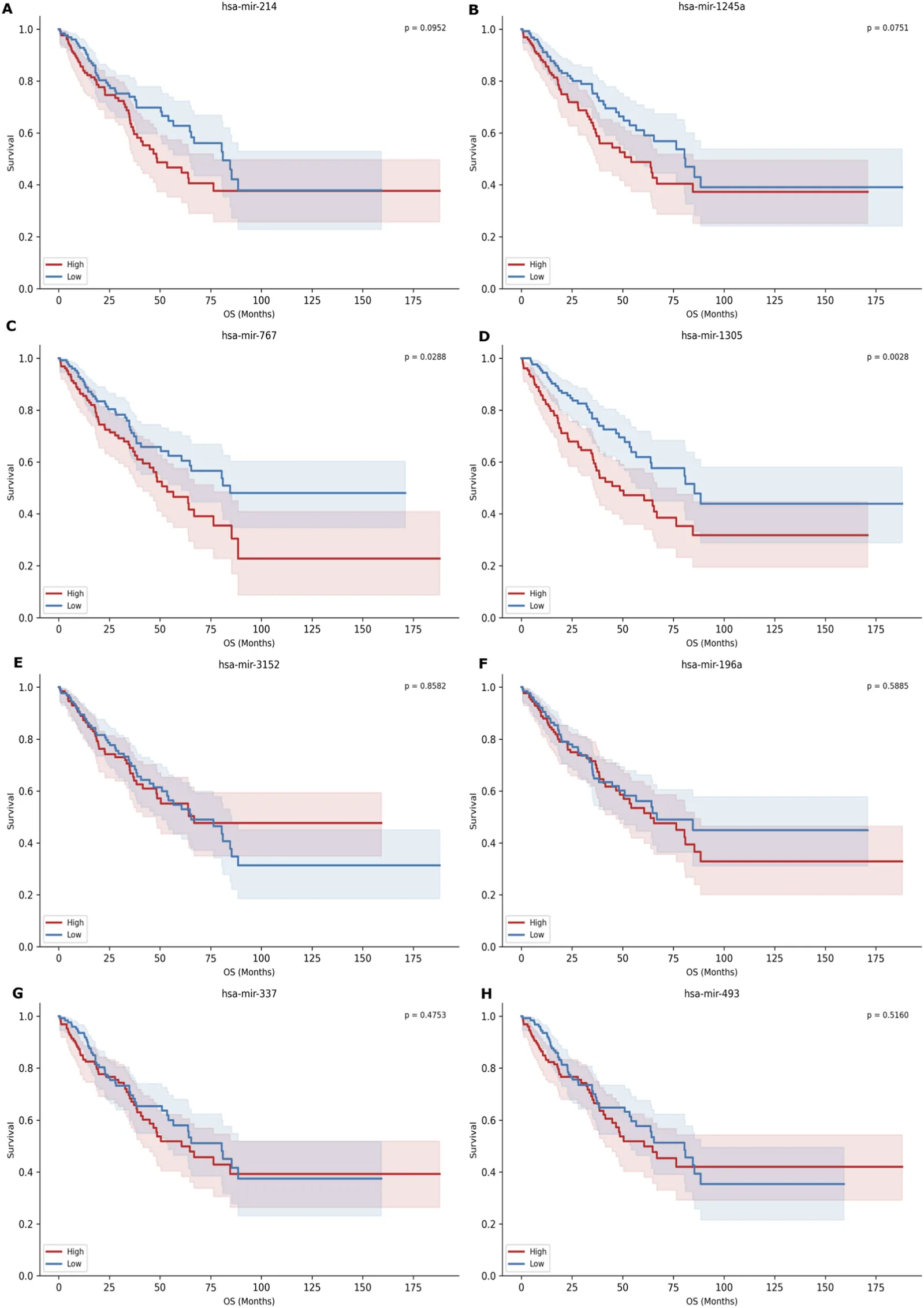
Kaplan–Meier survival analysis of consensus miRNAs in sarcoma. Kaplan–Meier overall survival curves for sarcoma patients stratified by high versus low expression (median split) for each of the eight consensus miRNAs. Survival probability is plotted over time (months), with shaded areas representing 95% confidence intervals. Log-rank p-values are indicated for each comparison. Part **(A-H)** represent the Kaplan–Meier graph of miR- 214, 1245a, 767, 1305, 3152, 196a, 337 and 493 respectively.

After BH correction, only one miRNA demonstrated a statistically significant association between expression level and overall survival. MiR-1305 showed the strongest prognostic association across the entire candidate set (log-rank p = 0.003; BH-adjusted p = 0.024). High expression of miR-1305 was associated with markedly shorter overall survival compared with the low-expression group (median OS approximately 48.55 months versus 85.38 months in the high and low groups, respectively), representing a difference of approximately 3 years in median survival.

MiR-767 showed a nominally significant log-rank result (p = 0.029); however, this did not survive BH correction (adjusted p = 0.116). Nonetheless, miR-767 was upregulated in sarcoma relative to normal mesenchymal tissue. MiR-214 and miR-1245a likewise showed trends toward inferior survival in the high-expression group (log-rank p = 0.095 and p = 0.075; BH-adjusted p = 0.190 for both), with median OS differences of approximately 32.4 and 26.8 months favouring the low-expression group. Both miRNAs were among the most significantly upregulated in sarcoma versus normal mesenchymal tissue.

In contrast, hsa-miR-3152, hsa-miR-196a, hsa-miR-337, and hsa-miR-493 showed no significant or near-significant survival differences between expression groups, suggesting that while these miRNAs contribute to the sarcoma miRNA anomaly signature as detected by the Isolation Forest framework, their expression levels do not stratify overall survival within the sarcoma cohort at this sample size.

## 4 Discussion and conclusion

This study asked whether the differences separating sarcoma from other cancer lineages at the level of microRNA expression are biologically meaningful rather than incidental, and whether any such biological signal carries clinical relevance. Three strands of evidence, considered together, are consistent with a biological signal. First, the sarcoma cohort was consistently separable from three developmentally distinct reference lineages using models that never saw sarcoma samples during training. Second, a subset of the consensus miRNAs driving this separation were also differentially expressed relative to normal mesenchymal tissue, distinguishing candidates whose anomaly signal is anchored in a genuine departure from the non-malignant state from those that may instead reflect retained lineage identity. Third, miRNA miR-1305 showed an association with overall survival. Taken together, these layers of evidence suggest that the consensus panel does not simply mark sarcoma as generically “different” from other tumours, but may capture, at least in part, biology relevant to mesenchymal identity, malignant transformation, and clinical behaviour—while stopping well short of establishing causal or clinical validity, for the reasons discussed below.

The graded pattern of discrimination across reference lineages (AUC-ROC 0.858–0.963)— strongest against neurological tissue, intermediate against haematological tissue, and weakest against epithelial tissue—is consistent with the hypothesis that developmental distance from the mesenchymal lineage shapes the miRNA landscape separating sarcoma from other cancers. Among the consensus panel, miR-214, miR-767, and miR-1305 are of particular interest because they combined high SHAP importance, significant overexpression relative to normal mesenchymal tissue, and, for miR-1305 an association with survival. miR-214 has been reported to promote proliferation and invasion in several tumour types via suppression of PTEN and downstream activation of PI3K–AKT signalling (Liu et al., 2015), (Liu et al., 2017), (Han et al., 2020), a pathway that was independently enriched in the present analysis and offers external, if indirect, support for the biological plausibility of this axis in sarcoma. miR-1305 has been linked to regulation of MDM2 in lung cancer and reported as a candidate prognostic marker in multiple myeloma (Cai et al., 2019), (Lee et al., 2021), (Zhang et al., 2020). These parallels in other tumour types do not establish that the same mechanisms operate in sarcoma; rather, they indicate that the candidates identified here are biologically plausible and merit direct experimental evaluation, not that they are confirmed sarcoma oncomiRs. The convergence of KEGG and Reactome enrichment on PI3K–AKT–mTOR signalling, p53-dependent DNA damage response and senescence, cell cycle control, and extracellular matrix/adhesion remodelling suggests that the consensus miRNA panel, considered collectively, could plausibly reinforce a transcriptional programme compatible with genomic instability and a dedifferentiated, invasive mesenchymal phenotype—features long associated with high-grade sarcoma biology (Renner et al., 2013). Through LDA-based analysis, we were able to identify Index 5, which was a set of five microRNAs mir-196a, hsa-mir-767, mir-1245a, mir-1305 and mir-493. This concordance is an encouraging computational result that supports the generalizability of the underlying miRNA signal beyond the discovery cohort. This also gives us future a direction that individual microRNA might not be the answer to microRNA biomarker, but a set of microRNAs that has underlying intertwined regulation.

The broader aim of this study was to come up with a framework that would assist biomarker discovery process in rare malignancies. Through this study we introduce a novel, explainable microRNA biomarker discovery framework that leverages anomaly detection to identify biologically meaningful regulatory signatures without relying on conventional differential expression or supervised classification approaches. By integrating unsupervised machine learning, normal tissue validation, and functional interpretation, the framework provides a generalisable strategy for distinguishing lineage-associated molecular identity from oncogenic dysregulation. Importantly, because the approach is agnostic to cancer type and molecular feature, it can be readily extended to other rare malignancies where limited sample availability constrains traditional biomarker discovery, as well as adapted to alternative high-dimensional molecular data, including mRNA, DNA methylation, proteomic, metabolomic, and multi-omics datasets. As such, this framework represents a broadly applicable computational strategy for discovering robust, interpretable biomarkers and uncovering disease-specific regulatory programmes across diverse biological contexts.

## 5 Limitation and future direction

However, several limitations of this study should be considered when interpreting its findings. Firstly, every result reported in this study rests entirely on computational and bioinformatics analysis of publicly available expression data. While this enabled access to sample sizes that would be otherwise difficult to attain in sarcoma, it also introduces potential batch effects, incomplete or heterogeneous clinical annotation, and cross-platform variability between RNA-sequencing and microarray-derived expression data. Although cross-platform normalisation was applied prior to validation, residual technical variation cannot be entirely excluded. Consequently, the consensus miRNAs (especially Index 5) though promising candidates supported by convergent, internally consistent computational evidence, should be put through experimental validation before it directed for clinical application. As with the discovery-cohort AUC in comparable index studies (e.g., Asano et al., 2019), the training-set LOOCV AUC reflects performance within the same cohort used for panel selection; the external cohort AUC is the primary evidence of generalizability. A major limitation to this study was that the matched normal reference tissue was defined only for the three dominant TCGA-SARC subtypes (leiomyosarcoma, liposarcoma, myxofibrosarcoma); since other represented subtypes were proportionally low, we decided not to provide a dedicated matched normal comparator to reduce bias, and findings for these subtypes should be interpreted cautiously. Since, the normal controls were split by accession so that every series contributed to both the training and validation pools, the validation is therefore an external sarcoma test set evaluated against held-out pooled controls and it is not a fully independent external cohort. Also, normal samples from GSE36982 were only used for normalisation; they were excluded during validation to prevent data leakage. miR-3152 was one of the consensus microRNAs, and the reason we could not use it during our validation process and differential expression analysis was not enough samples having the expression values of miR-3152. This does not reduce the credibility of miR-3152 but a limitation beyond our control. Thus we had continued using mir-3152 in other analysis.

Secondly, TCGA-SARC spans across many histological subtypes, of which leiomyosarcoma and liposarcoma together account for the majority of samples in this cohort. The consensus miRNA panel and the associated pathway and survival findings were derived from this pooled cohort and may consequently be disproportionately shaped by the biology of these dominant subtypes rather than reflecting a signature common to sarcoma as a class. This is a meaningful limitation for generalizability: a panel that performs well when leiomyosarcoma and liposarcoma dominate the training data may not fully transfer to less-represented subtypes, such as synovial sarcoma, rhabdomyosarcoma, or undifferentiated pleomorphic sarcoma, which differ substantially in driver biology, cell of origin, and clinical behaviour from the smooth-muscle and adipocytic lineages that anchor much of the present cohort. The subtype-matched selection of normal reference tissue—myometrium for leiomyosarcoma, adipose tissue for liposarcoma, fibroblasts for myxofibrosarcoma—was a deliberate attempt to align each subtype with an appropriate developmental counterpart, but it does not fully resolve this underlying imbalance on the tumour side of the cohort.

Finally, this study evaluated a single anomaly detection algorithm (Isolation Forest). While this choice was motivated by its native one-class formulation, compatibility with TreeSHAP and superiority in handling multi-modal data sets, alternative anomaly detection approaches (e.g., one-class support vector machines, autoencoder-based reconstruction error, or deep isolation forests) may offer complementary sensitivity to different modes of sarcoma-specific deviation and represent a natural avenue for methodological comparison.

Future work should prioritise prospective, multi-institutional validation of the Index 5 panel, ideally incorporating a clinically deployable assay format (e.g., targeted qPCR). Extending the anomaly detection framework to individual sarcoma subtypes, contingent on adequate subtype-specific sample sizes, would help determine whether the consensus signature reported here reflects a pan-sarcoma phenomenon. Cox analysis was not performed since clinical data do not permit a reliable adjusted analysis. Functional validation of the proposed hub target genes, particularly those implicated in the PI3K-AKT-mTOR and p53-senescence pathways, would help establish causality rather than association. More broadly, given that the core contribution of this work is methodological, an important next step is to test the transferability of this anomaly detection and explainable-AI framework to other rare cancer types beyond sarcoma, which would substantiate its utility as a generalisable solution to the small-sample-size problem in rare-disease biomarker discovery. While this study lacks experimental and clinical validation, the broader aim of this study was to come up with a framework that would assist and speedup the exploratory candidate biomarker discovery process in rare malignancies. But we request readers discretion while extrapolating the results of this study and would encourage future studies focusing on the experimental validation which would make the use of this framework truly worthy.

## Data Availability

Publicly available datasets were analyzed in this study: Internal Sarcoma cohort: TCGA-SARC; External Sarcoma cohort:GSE36982; Epithelial (Breast: TCGA-BRCA, Lung: TCGA-LUSC/LUAD, Colorectal: TCGA-COAD/READ), Haematological (Leukaemia: TCGA-LAML/AML/ ALL-P2, Lymphoma: TCGA-BLGSP), Neurological (Glioma: TCGA-LGG); Normal sample(TCGA-UCEC, GSE45363, GSE25470, GSE84971, GSE18470, GSE159959). The study IDs and sample IDs used in this study and their source can be found here: https://github.com/Mritunjoy-Dey/Sarcoma-miRNA-anomaly-detection/blob/main/All_Samples_Study_IDs.csv.
