# Supplementary material for: Cross-lineage anomaly detection of microRNA expression: a framework for deriving exploratory candidate biomarkers in sarcoma
Source: Front Genet. 2026 Oct 12;17:1907450. doi: 10.3389/fgene.2026.1907450 (PMC13696034; doi:10.3389/fgene.2026.1907450)
Supplement: Supplementary file 1 [file DataSheet1.pdf]

Fig S1: PCA plot, Epithelial cohort PCA

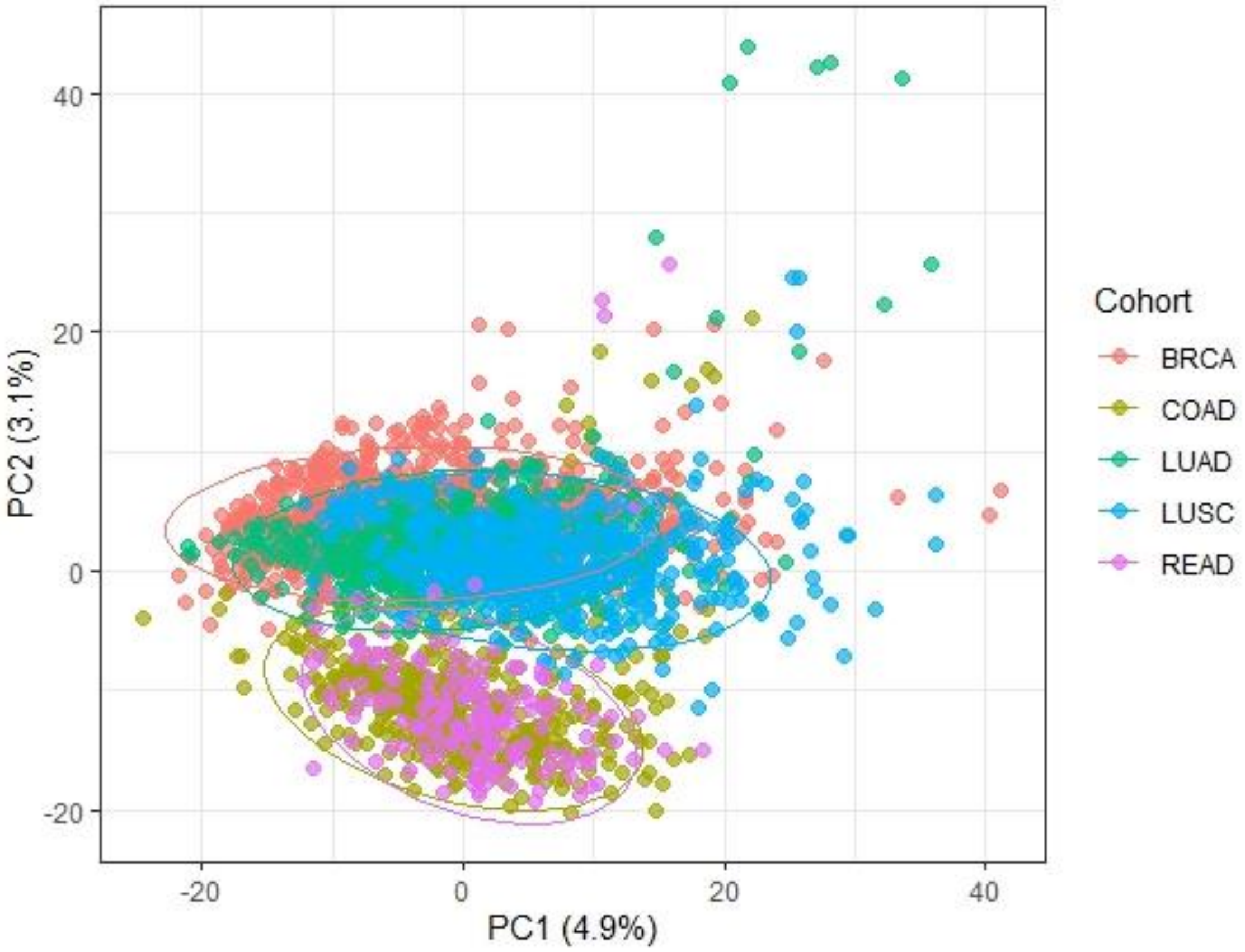

Fig S2: PCA plot, Haematological cohort PCA

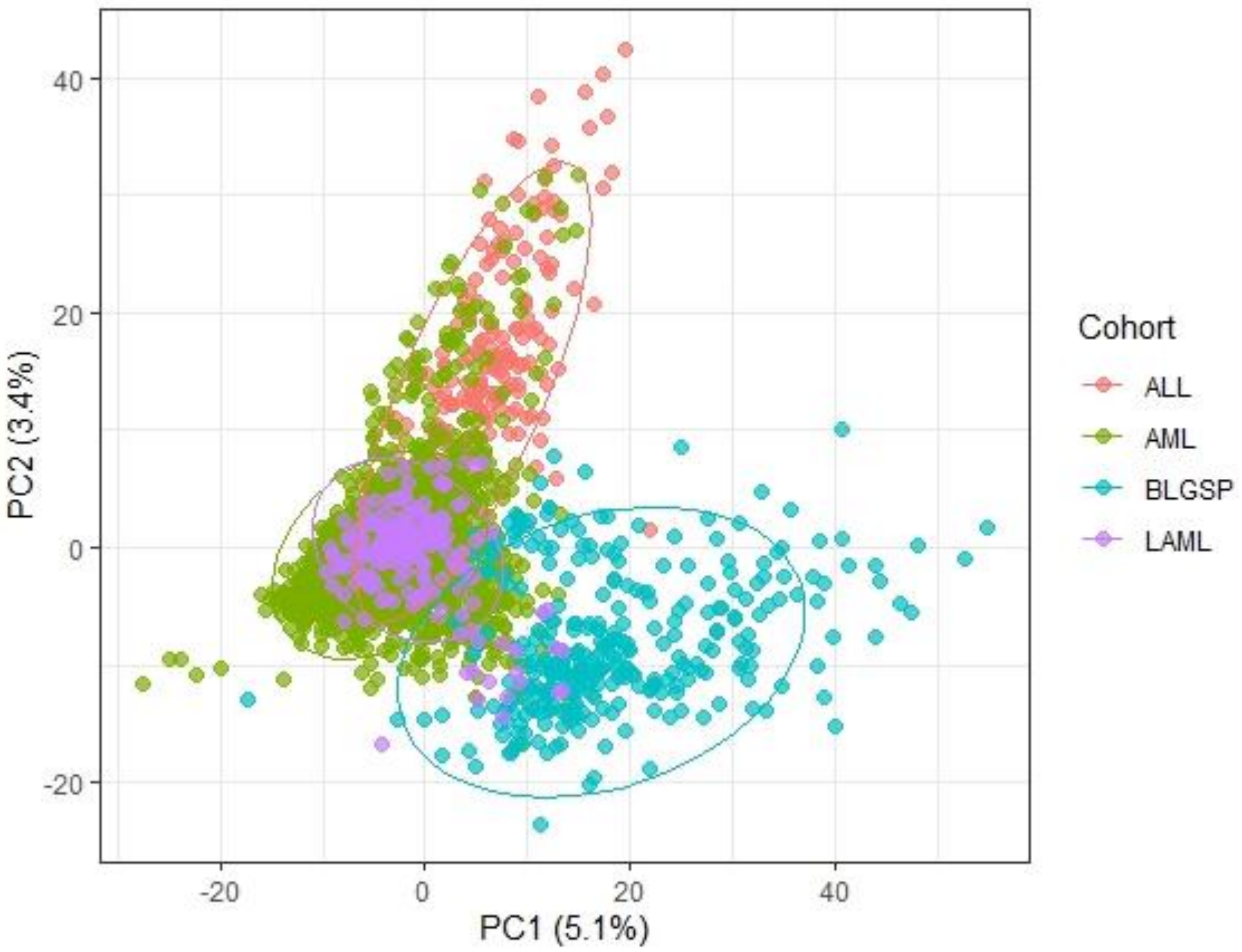

Fig S3: PCA plot of normal samples before and after normalization

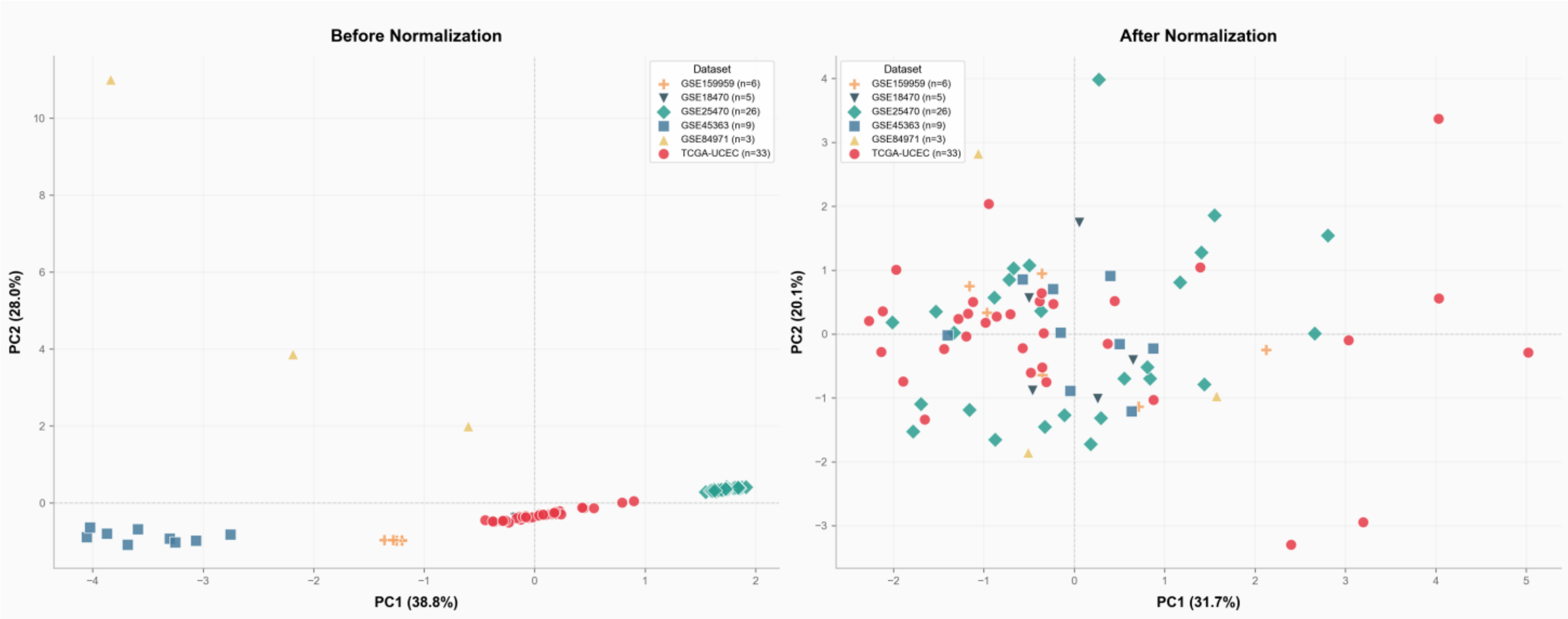

Table SI:SHAP Importance of top 30 microRNA candidates in each cohort comparison

S.No	Epithelial vs Sarcoma	SHAP importance	Haematological vs Sarcoma	SHAP importance	Neurological vs Sarcoma	SHAP importance
1	hsa-mir-200a	0.056236647	hsa-mir-142	0.030066366	hsa-mir-9	0.069296055
2	hsa-mir-200c	0.050950504	hsa-mir-196a	0.027032084	hsa-mir-153	0.066443919
3	hsa-mir-200b	0.050518713	hsa-mir-15a	0.025070848	hsa-mir-199b	0.051930743
4	hsa-mir-429	0.042165615	hsa-mir-19b	0.02143531	hsa-mir-214	0.038907712
5	hsa-mir-141	0.032155216	hsa-mir-17	0.020905173	hsa-mir-488	0.037511649
6	hsa-mir-182	0.02786148	hsa-mir-204	0.020203824	hsa-mir-199a	0.03567646
7	hsa-mir-203a	0.027431524	hsa-mir-7	0.01934682	hsa-mir-196a	0.03390983
8	hsa-mir-375	0.024572497	hsa-mir-891a	0.019199648	hsa-mir-338	0.033872163
9	hsa-mir-183	0.023593147	hsa-mir-1262	0.018887643	hsa-mir-338	0.032845145
10	hsa-mir-96	0.019960787	hsa-mir-20a	0.018314103	hsa-mir-1245b	0.030438919
11	hsa-mir-369	0.017094696	hsa-mir-19a	0.01777196	hsa-mir-3934	0.027398676
12	hsa-mir-4421	0.017044596	hsa-mir-767	0.016319753	hsa-mir-675	0.026882854
13	hsa-mir-655	0.01654694	hsa-mir-25	0.016282221	hsa-mir-1305	0.026711993
14	hsa-mir-379	0.016003085	hsa-mir-942	0.016245859	hsa-mir-3199	0.022582213
15	hsa-mir-432	0.015843633	hsa-mir-105	0.016230311	hsa-mir-10a	0.02160986
16	hsa-mir-154	0.015431752	hsa-mir-3615	0.015980505	hsa-mir-511	0.020155495
17	hsa-mir-337	0.015111141	hsa-mir-146a	0.015326587	hsa-mir-1245a	0.020017226
18	hsa-mir-136	0.014495468	hsa-mir-766	0.015242719	hsa-mir-493	0.019568707
19	hsa-mir-1185	0.014263095	hsa-mir-185	0.015154418	hsa-mir-3154	0.019034163
20	hsa-mir-299	0.014188791	hsa-mir-1305	0.014943643	hsa-mir-155	0.018317757
21	hsa-mir-487b	0.014061555	hsa-mir-1245a	0.014664072	hsa-mir-3200	0.018005059
22	hsa-mir-329	0.013931739	hsa-mir-181a	0.014299118	hsa-mir-767	0.017682067
23	hsa-mir-127	0.013883835	hsa-mir-888	0.014212659	hsa-mir-337	0.017158309
24	hsa-mir-889	0.013554101	hsa-let-7c	0.014163518	hsa-mir-581	0.017026707
25	hsa-mir-380	0.013453562	hsa-mir-892a	0.014133142	hsa-mir-181c	0.016483639
26	hsa-mir-376c	0.013431393	hsa-mir-652	0.014065007	hsa-mir-145	0.01616525
27	hsa-mir-668	0.013403506	hsa-mir-337	0.013930579	hsa-mir-196b	0.015937005

28	hsa-mir-493	0.013117849	hsa-mir-3152	0.013891594	hsa-mir-1247	0.015922841
29	hsa-mir-134	0.013076926	hsa-mir-30e	0.013887753	hsa-mir-4772	0.015638564
30	hsa-mir-654	0.012966268	hsa-mir-214	0.013789025	hsa-mir-3152	0.015464608

Table SII. Summary of the scale of the data, the platform on which they were processed and for which datasets log2 transformation was applied

Dataset	Platform	Deposited scale (GEO/submitter)	Log2 transform applied	Normal reference source
GSE159959	Exiqon miRCURY LNA microRNA Array (7th gen)	Log-scale (per GEO submitter)	No	GSE159959
GSE18470	Agilent-019118 Human miRNA Microarray 2.0	Log-scale (per GEO submitter)	No	GSE18470
GSE25470	Affymetrix Multispecies miRNA-1 Array	Log-scale (per GEO submitter)	No	GSE25470
GSE36982	Illumina Human v2 miRNA beadchip	Log2, quantile-normalized (per GEO submitter)	No	GSE36982(normal samples)
GSE45363	Exiqon LNA probe array v10	Linear intensity (per GEO submitter)	Yes	GSE45363
GSE84971	nCounter Human miRNA Expression Assay v2	Linear raw counts (nCounter)	Yes	GSE84971
TCGA-SARC	Illumina HiSeq RNA-seq	Normalized, log-scale (Xena RNA-seq)	No	TCGA-UCEC
TCGA-UCEC	Illumina HiSeq RNA-seq	Normalized, log-scale (Xena RNA-seq)	No	TCGA-UCEC
